# Challenges and Facilitators During Transitions from Adolescent Medium Secure Units to Adult Services in England: Interviews with Mental Healthcare Professionals

**DOI:** 10.1007/s10488-021-01115-9

**Published:** 2021-02-24

**Authors:** Maria Livanou, Sophie D’Souza, Rebecca Lane, Breanna La Plante, Swaran P. Singh

**Affiliations:** 1 Department of Psychology, School of Law, Social and Behavioural Sciences, Kingston University, Kingston Upon Thames, London, Surrey, KT1 2EE UK; 2 University College London, Gower Street, London, WC1E 6BT UK; 3 Anna Freud National Centre for Children and Families, The Kantor Centre of Excellence, 4-8 Rodney Street, London, N1 9JH UK; 4 Division of Health Sciences, Mental Health and Wellbeing, Warwick Medical School, Coventry, UK; 5 Birmingham and Solihull Mental Health NHS Foundation Trust, Birmingham, UK

**Keywords:** Transitions, Mental healthcare professionals, Adolescent secure services, Emotional readiness

## Abstract

Young people moving from child and adolescent secure hospitals present with complex needs and vulnerabilities and are more likely to experience poor transition outcomes. Previous research has indicated the presence of several risk factors in periods of transition, such as poor liaison among services, lack of proper planning, shortage of beds in adult services, multiple transitions and lack of emotional readiness. However, little evidence exists about the processes and outcomes of transitions from adolescent secure services to adult settings. This study aims to bridge the gap in the existing literature by exploring the views and experiences of key professionals involved in the transition process from six adolescent medium secure units to nine adult secure and community services in England. Thirty-four key workers from 15 child and adolescent (*N* = 21) and adult (*N* = 13) forensic hospitals were interviewed to provide information about potential barriers and facilitators to transitions. Face-to-face semi-structured interviews were conducted between January 2016 and December 2017. Thematic analysis was used to identify challenges and facilitators to transitions. Three primary themes were identified: (1) transition processes and preparation; (2) transition barriers and challenges; (3) success factors to transition. Key differences in adult and adolescent service care-models and lack of emotional and developmental readiness to moving onto adult-oriented settings constitute major barriers to positive transition outcomes. Practice and policy implications are considered to address the need for service transformations.

## Introduction

Transitions across mental health services in the UK are considered problematic due to a number of organisational and infrastructural challenges, such as transition delays, lack of bed availability and poor liaison between child and adult mental health services (Singh et al. 2010). There is substantial evidence that transitions from paediatric services to adult care are linked to an exacerbation of medical and mental health symptoms (Campbell et al. 2014). Previous research has additionally classified transitions as a global priority for chronic conditions, including mental health (Campbell et al. 2014). Subsequently, there has been an urge in recent years for mental health services to aim for a ‘better’ interaction with the legal framework (Hales et al. 2019).

Over the past two decades, transition of care from child and adolescent mental health services (CAMHS) to adult-centred settings has received global attention. Adolescence comprises a high-risk period for the emergence of mental disorders due to cognitive, biological, social, developmental and emotional changes (Singh et al., 2008). Young people in contact with mental health services experience both healthcare and institutional transitions. It is well established in the extant literature that poorly planned and executed transitions may have an adverse impact on young people’s mental health (Livanou et al. 2020). Young people in contact with youth justice services and secure care present with multiple complexities such as high-harm, high-vulnerability, as well as high-risk, and the implications of poor transition outcomes extend to poor mental health and increasing reoffending rates upon returning to the community (Hales et al. 2019). Systematic continuity of care and follow-up are not currently standard practice by child and adolescent services. Upon transitioning to adult mental health services, service disruptions therefore result in a lack of follow-up of mental health outcomes for this group.

Service provision for young people in mental health services during transition periods fails to adequately meet precipitating and ongoing needs, particularly for those with comorbid mental health difficulties (Singh et al. 2005). Previous research has identified existing barriers to successful transitions from CAMHS to adult mental health services (AMHS) such as waiting time, rigid referral criteria, continuity of care, age cut-off, lack of communication between services, lack of understanding regarding developmental needs, abrupt transitions and lack of staff training (Singh et al. 2010). Historically there has been a gap between the two services regarding treatment approaches and care priorities. This gap in philosophy pertains to AMHS adopting a more unimodal approach towards the service user and CAMHS using a multimodal line, which integrates biological, psychological and developmental factors towards treatment and rehabilitation (Paul et al. 2013).

There are very few studies on transitional care amongst young people who offend, and these only include small samples. In England and Wales, young people committing an offence can be transferred to the children and young people’s secure estate (CYPSE), comprised of Young Offender Institutions (YOI), Secure Children’s Homes (SCH) and Secure Training Centres (STC). In cases where young people present with persistent mental health problems, which cannot be managed in the secure estate, they will be moved to adolescent secure hospitals to receive appropriate treatment. However, longitudinal outcomes of this group’s transitional experiences once they reach 18 years and are transferred to adult services are not known.

The Care Quality Commission (2019) reported on the detrimental effects of long-term segregation on young people recently transferred to adult forensic services. This report highlights the difficulty for particularly vulnerable young people with neurodevelopmental needs to adjust to adult wards. Young people with autism and/or learning disabilities experience greater distress in unfamiliar environments, which may explain higher risk presentation during transition periods (Hollins 2019). Furthermore, during transition periods, service provision declines, increasing the gap between child and adult services. The current divide perpetuates the problematic nature of transitions due to disconnected services (Signorini et al. 2018). Research findings echo the need to improve transition outcomes for young people accessing mental health services and to design age-appropriate services with the implementation of specialised transition programmes.

There is very limited evidence about the views and transition experiences of mental healthcare professionals in forensic settings (Hales et al. 2019). This study employed face-to-face semi-structured interviews to bridge the current gap in the literature by exploring the views and experiences of key professionals involved in the transition process from six adolescent medium secure units to nine adult secure and community services in England. This research aimed to understand and reflect on the experiences of mental healthcare professionals to improve future transition processes and outcomes and inform policy as young people move from adolescent forensic services to adult-oriented settings. The interviews aimed to identify barriers and facilitators in the transition process.

## Methods

### Research Design

The technique of purposive sampling was applied, recruiting on the basis of staff availability and keenness to taking part. We aimed for a representative sample and, therefore, we targeted mental healthcare professionals from all six nationally funded medium secure services for adolescents in England and selected adult services based on young people’s transition destination at the time of the interviews (six months December 2016–June 2017).

### Participants

Thirty-four key workers (psychiatrists, psychologists, nurses, occupational therapists, social workers, healthcare support workers, and family therapists) were recruited from fifteen services (see Table 1). Seventy-four percent was from white ethnic background, 15% were South-Asian and 3% were Black. Thirty-eight percent were females from which 24% were psychiatrists, 31% psychologists, 15% nurses, 15% social workers and 15% family therapists (see Table 1). There were no male social workers, nurses or family therapists. Certain types of professionals were underrepresented, such as occupational therapists and nurses. The most common type of involved professionals were psychiatrists due to staffing levels. Nurses had high clinical caseloads and, therefore, were more difficult to recruit. Staff specialist numbers varied across hospitals; however, all national services offered a range of activities through art therapy or occupational therapy. One adolescent ward did not have an occupational therapist and none of the units had a primary mental health practitioner or psychodynamic psychotherapist. Two hospitals did not have a family therapist. The average staff ratio in adolescent medium secure services is 0.54 (*SD* = 0.32). Each of the six adolescent services had an average of two psychiatrists, two psychologists and 25 nurses, one social worker, one occupational and one family therapists. A previous scoping exercise on staff ratio showed that the average staff ratio was 38 and the recruited sample represented about 11% of the overall staff population in adolescent secure services (Livanou et al. 2020). This information is not available for adult services considering the variety of services involved and the lack of a relevant scoping exercise.

**Table 1:** Types of healthcare professionals interviewed across mental health settings

Interviewees	Number	Percentage
Child and adolescent key workers	N	%
Psychiatrists	9	26
Psychologists	5	15
Social workers	2	6
Nurses	2	6
Family therapists	2	6
Occupational therapist	1	3
Adult key workers		
Psychiatrists	9	26
Psychologist	1	3
Social worker	1	3
Nurses	1	3
Health support worker	1	3
Sex		
Females	13	38
Males	21	62
Ethnicity		
White	25	74
Black	1	3
South Asian	8	3
Length of time in current role	Median = 18 months (6–36)	

### Recruitment and Consent

An initial visit was conducted by the principal researcher (ML) to all adolescent and adult services to introduce the research and meet the clinical teams. The local collaborator from each service facilitated this process by arranging an introductory meeting with the multidisciplinary (MST) teams to explain the objectives of the study. Recruitment occurred in groups. Any mental health professional working in the service for a minimum of six months was invited. The participants had been in their current role between 6 months and 3 years. Initially, it was expected that 20–25 mental healthcare professionals would be recruited in total to meet the requirements of the study. The recruitment of more mental healthcare professionals was necessary to reach theoretical saturation but also to include a wide range of views from a diverse body of professionals within mental health settings. Forty-two potential participants were approached and had expressed their interest to partake in the study. However, eight mental healthcare providers (from forensic adolescent services) were not available for an interview considering high clinical caseloads and being part-time staff upon following visits. Twenty-one semi-structured interviews were conducted with mental healthcare professionals from child and adolescent forensic services and thirteen interviews with mental healthcare professionals from the adult receiving services.

### Recruiting Sites

In England there are currently six adolescent medium secure units nationally funded and distributed across six geographical locations. All six units were included and visited to recruit participants for the study. Additionally**,** nine different adult services across England were visited to follow up with adult key workers including nine adult forensic secure hospitals and two community supported accommodation settings, identified by responsible clinicians (RCs) from adolescent services. All interviews were individual and took place in mental healthcare professionals’ private offices or other allocated spaces such as interview rooms. The participants were not compensated.

### Procedures

All participants provided written informed consent. The principal researcher (ML) conducted all the interviews face-to-face between January 2016 and December 2017. The interviews took place in secure services and lasted between 20 and 90 min (*M* = 40, *SD* = 19.86). The length of time for interviews was adjusted to individual needs. Interview time varied depending on availability, clinical caseloads, and staff ratio. Certain participants were more descriptive and required more interview time to delineate the transition process. All identifying information were removed and kept anonymous. Detailed field notes and reflective summaries were written post-interviews. Ethical approval for the study was obtained from the Health Research Authority (HRA) as part of a larger educational project and qualified as an NIHR CNS Portfolio study in January 2016.

### Measures

The TRACK interview topic guide, which was part of a larger national study about transitions from mainstream CAMHS to adult mental health services, was used with some minor alterations that pertained mainly to the forensic nature of the services included in this study (Singh et al. 2008). The TRACK topic guide for child and adolescent and adult healthcare providers covered questions and probes relevant to transition preparation and discharge policies, infrastructural weaknesses, continuity of care, joint and parallel working between services: (1) What is the frequency of team meetings between CAMHS and adult services? (2) What is the process of collaborative decision making? (3) What are the greatest challenges to achieving successful transition in the way services are currently organised? These items were relevant to the present study considering the role of joint services and shared decision making. This study’s topic guides for child and adolescent services targeted factors influencing continuity of care, access to AMHS, effective communication, multiagency liaison, further recommendations for policy, resources to facilitate transition and continuity, and skills and training necessary to improve staff confidence in provision of effective transition and continuity of care to young people and carers. Topic guides for adult mental health services focused on organisational and structural difficulties in transition processes to shed light on the interaction between mental health care and continuity of care for young people. The interview guides were flexible and allowed participants to expand on important transition topics.

### Coders

The principal researcher had been trained in qualitative methods and analysis by attending a relevant course. The second coder (SS) is a clinical psychiatrist and researcher with extensive experience in qualitative coding and expertise in youth transitions.

### Codebook Development

The principal researcher developed a codebook including encoded and predetermined terms such as continuity/discontinuity of care, liaison among services, referral criteria, barriers/facilitators to transition, waiting time, staff training, structural/organisational barriers, service satisfaction, transition barriers/facilitators, family’s role, continuity/discontinuity of care, service coordination, and mental healthcare professionals’ flexibility (Singh et al. 2010). The a priori (literature driven) codes were aligned with themes elicited in the TRACK study and a peer-debriefing followed in which codes were discussed and adjusted to fit in forensic transitions. Similar codes to the TRACK project were identified in the current study including lack of staff training, delayed transitions, infrastructural weaknesses and joint working between services. The TRACK study has clearly established that these factors impede transition preparation and management (Singh et al. 2010). Emerging codes included being surrounded by older peers, shift in social status, risk presentation, Aftercare under the Mental Health Act.

This codebook was revised during the interview period and the codes were refined using a reflexive approach. Reflexive approach is an ongoing process employed by the researcher to facilitate careful examination of pre-existing assumptions and minimise researcher biases brought into analysis (Begoray and Banister 2010). Reflexivity allows self-awareness about motivations, actions, and power dynamics relevant to the research topic aiming to further readers’ understandings (Given 2008). Critical reflexivity aids the researcher to recognise the friction between personal involvement and detachment and enhances the validity of the research.

### Data Analysis and Interpretation

The interviews were audio-recorded, transcribed and analysed using thematic analysis (Braun and Clarke 2006). Four interviews were not audio-recorded due to ward restrictions and field notes were taken instead. Transcripts were analysed using MAXQDA software to identify emerging patterns and trends including differences, similarities, contradictions, repetitions, summaries, and use of language. Braun and Clarke (2006) literature- and data-driven six-step approach was followed to analyse the dataset. The principal researcher (ML) became familiar with the data and then transcribed the context and re-read the dataset multiple times. ML transcribed the data to develop a thorough understanding of the context, as suggested by Braun and Clarke (2006). The transcripts were checked against the audio-recorded interviews to ensure accuracy. Before coding, ML discussed and reflected on transition key themes with the mental healthcare professionals post-interview time to ensure credibility. Accordingly, any potential biases were minimised. Coding focused on reflecting the transition experiences of key workers. The detailed notes for the four interviews not audio-recorded were turned manually into a reflective summary which was imported to MAXQDA software where coding and thematic analysis were performed similarly to the recorded interviews.

**Fig. 1:**
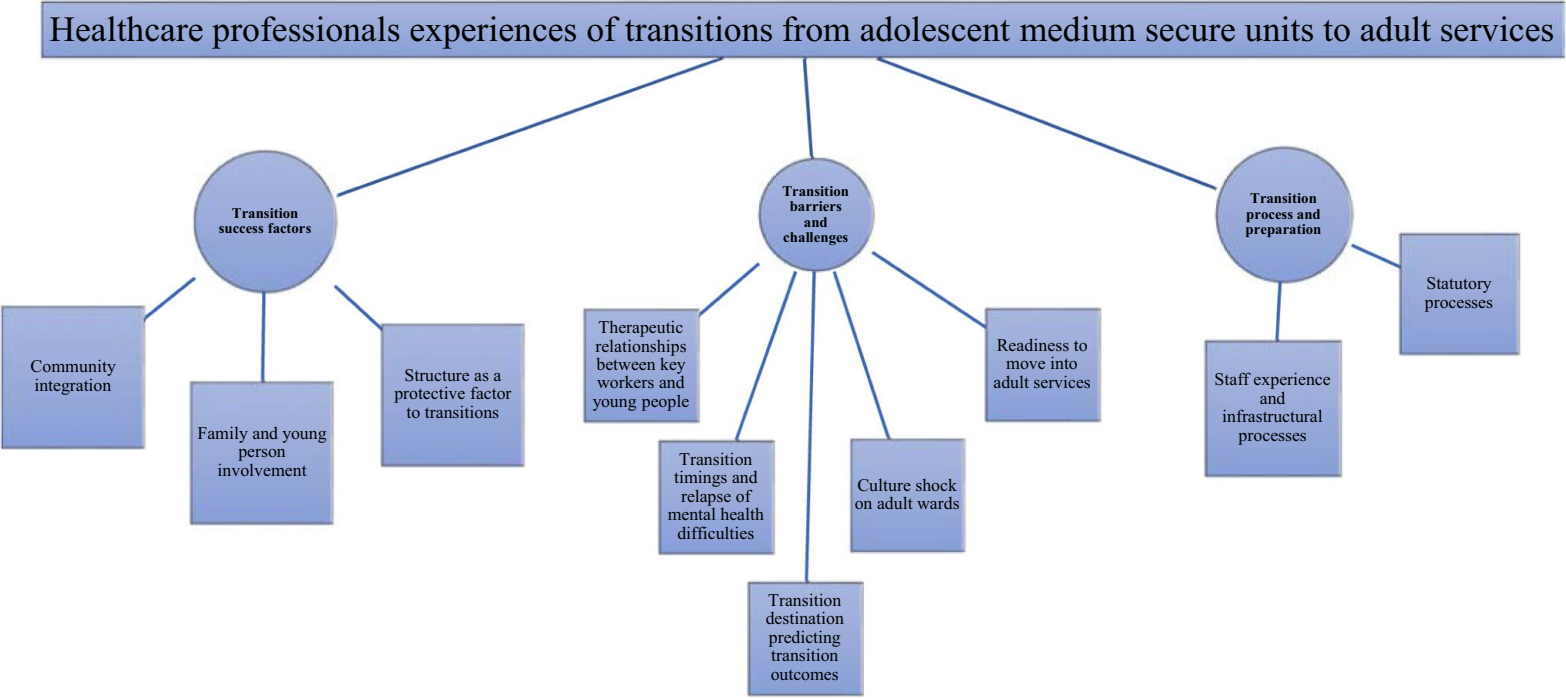
Diagram showing the interview topic (level 1), themes (level 2) and sub-themes (level 3)

ML conducted line by line coding independently by constant comparisons of similarities and differences in-between the 34 transcripts. Thirty percent of the transcripts were double coded independently by SS. Inter-rater reliability was 0.84. Inter-rater reliability was based on theme frequency and whether a theme was present or absent. Discrepancies were discussed between the two coders and resolved through reflecting on post-interview interpretation of the data with the participants. When the two coders agreed, the final definition of the code was assigned and determined. The results were discussed with 90% of the sample and reflected on their responses post-interviews to allow for credibility checks. The interpretation of the findings was also confirmed with participatory providers to check on accuracy of their experiences via supplementary visits to services-average five visits had taken place to each adolescent site-to meet with providers and two to adult sites. Overall, there was consistency across the interviews. We aimed for concept consistency between the researcher and the participants. The codes were turned into themes which portrayed the data. Next, the themes were reviewed checking for coherent patterns and refined by ML and SS ensuring theme definitions were consistent with the findings. Constant comparison between old and new themes took place with an ‘audit trail’, where all decisions and activities were documented to enhance trustworthiness. Finally, agreed themes were determined and linked to corresponding quotes. Following refinement, a thematic map was produced (Fig. 1). The content and clarity of sub-themes were reviewed multiple times to reach consensus. The analytical framework was based on latent analysis, as described by Braun and Clarke (2006), where an in-depth analysis takes place and aims to unravel latent content underlining the interviews (Braun and Clarke 2006). In this case, latent analysis was performed in the final themes and sub-themes to elaborate on the semantic context and the development of conceptualisations.

A *contextualist* approach was used as the main epistemological position, which lies in between constructionism and essentialism. From a constructionist perspective, secure hospitals are confined environments that can impact mental healthcare professionals’ views whereas an essentialist perspective is based on the objective reality of all participants. In the present study, we aimed to understand the experiences of key workers in relation to their context and individual circumstances.

## Results

Please see the results summary in Tables 1 and 2.

**Table 2:** Numbers per mental health profession recruited across 15 hospitals

Adolescent medium secure units	Psychiatrist	Psychologist	Social worker	Nurses	Family therapist	Health support worker	Occupational therapist
1	3	1	1				
2	1	1	1	1	1		1
3	2	1			1		
4	1	2					
5	1			1		1	
6	1						
Adult services							
1. High secure unit	2	1	1	1			
2. Medium secure unit	1						
3. Medium secure unit	1						
4. Low secure unit	1						
5. Medium secure unit	1						
6. Community support accommodation	1						
7. Low secure unit	1						
8. Low secure unit	1						
9. Community support accommodation						1	

### Overview of Themes

Three overarching themes were identified: (1) transition processes and preparation; (2) transition barriers and challenges; (3) transitions success factors (Table 3). All themes and sub-themes were identified in both adolescent and adult key-workers (see Fig. 1). Each of these themes are discussed and sub-themes expand on key ideas.

**Table 3:** Primary themes, subthemes and illustrative quotes

Primary theme	Subtheme	Illustrative quote
Theme 1: transition processes and preparation	Statutory processes	“We use the CPA and that involves the final meeting is a written note according to the Mental Health Act and that is the Section 117 meeting. So that’s the sort of formal procedure that supports transitions and that ensures that the way that in which the other units have to interact with us, and then other aspects of transition and transfer are to know on individual basis, depending on need and risk.” Psychologist 1 “The process of preparation each discipline will work with them on a discharge plan, they will work with them on what, it’s more about their communication. They will have a communication folder that describes who they are, what are the risk concerns, what they like and what they don’t like, what are the things they worked through and what are the things they need to work through, so that’s for them to take along with them.” Psychiatrist 1
	Staff experience	“It’s very difficult, very challenging. It’s easier going to an adult hospital from a commissioning point of view. And that also comes out quite a bit that you think of care coordinator. There’s always a gatekeeping assessment and then it depends on that but usually because it gets so much variable you usually have a quite good idea before they come for the gatekeeping assessment anyway. Care-coordinators you see them getting nervous when you talk about what placement they have in the community, the forensic, and the offending and the risk point of view but also I think it’s the logistics and sorting it out.” Occupational Therapist “When young people come to us they are aware that our service is designed for 12 to 18 year olds. So that’s established to begin with. So we have that discussion early on so they know that the part of getting support from us is planning how they can maintain their recovery and continue the gains they have made when they are actually part of the community. I think rather than breaking relationships it is allowing clear communication from the start that the work that we can do with the young person is limited. But also reassuring them that there is scope for ongoing support and the earlier that we start that the better.” Psychologist 2
Theme 2: transition barriers and challenges	Therapeutic relationships	“I think the tribunal process is important but sometimes it’s very stressful for patients. So, if they appeal against a section, the tribunal is quite a formal thing in the court, how long they’ve been in hospital for, they are a stressful experience for the patient. The consultant, the Responsible Clinician, has to argue for the continued detention of the patient and, usually, the patient is asking to take them off the section. So, it can become quite adversarial, it can worsen the relationship between the consultant and the patient.” Psychiatrist 5 “We have a lot of young people who are admitted to the service relatively late at 17.5 plus and is very difficult to meet their needs when suddenly they are on their 18^th^ birthday and just started to engaging in formal treatment and make relationships with staff and the pressure is moving them to another service, I think what this inevitably does is extending their stay in hospital.” Psychiatrist 2
	Transition timings	“So X, he’s 18, and was accepted by an adult secure hospital, sometime ago, but they don’t have a bed. It’s completely clocked up the system. So he’s waiting and that is very bad for him. […] they can’t tell us when a bed may be available and we have serious problems for X because he started disengaging from our service. He’s sort of frustrated that he’s still here with the kids, as he reasons. So yes he’s sort of stuck at the moment until a bed comes up.” Psychiatrist 4 “There’s a lot of people who relapsed they got to the point they were ready for discharge but it took that long that they ended up back to square one; they relapsed.” Nurse 1
	Transition destination	“The extended period of time, especially, when we don’t have an end date, because the patient becomes very demoralised and destabilised. In fact, the nursing staff, the whole team becomes kind of desponded about. You know they need to move on, we’ve done as much as we can…and you can have aberrant behaviours re-emerging stuff. When they don’t know when they’re going that causes the most problems.” Psychiatrist 5 “When the risk is high, they will be transferred without these visits but they will be provided information about the service…They wouldn’t know the date of transfer.” Psychiatrist 1
	Culture shock	“When people go to from here to inpatient units, I think it’s a big shock to the patients because they go from this very nurturing environment where even though is quite chaotic sometimes here, I think it’s much more ordered than an adult unit. So we get a lot of our patients smashing things, shouting, crying; they are very emotional. But we can contain that to an environment where is much lower staffing level and there’s much larger groups of patients and the patients are not supervised for much longer.” Psychiatrist 9
	Readiness to move onto adult services	“They get so frightened and cowered… then you’re going into an adult service where there’s going to be people in their 30 s and 40 s and maybe in their 50 s on the same ward. It must be terrifying for the young people and terrifying for the families.” Social Worker “I think it’s a very tricky situation because just on the previous day of their 18th birthday they were just 17 and the day after they become adults, is there any change overnight? I don’t think so. It’s a gradual process. I would think there should be an intermediate service, like service for 18 to 25 s.” Psychologist 3
Theme 3: transition success factors	Community integration	“If you got a good care-coordinator and if you are linked with local services from the onset, that really helps.” Psychologist 4 “All the young people are under the Mental Health Act so they’re all depending on which section they are on but they all have to be discharged from a section of the Mental Health Act conditionally. Most of them have got an entitlement to Aftercare under Section 117. I guess on the whole the Mental Health Act plays a positive role, probably. It gives young people that are very restricted and being looked after, in some sense, it gives them some protections in that and some rights and it does give them some follow-up entitlement.” Family Therapist 1 “The Mental Health Act is useful—you got to discharge them and you still have treatment obligations carried out, which means they are assured to get follow up. I can’t think of a situation, where I said God I have to detain them under the Act.” Psychiatrist 7
	Family and young person involvement	“We do have family therapy, we do welcome meetings, we do open days for the parents to get them engaged and to give them more understanding and more help.” Psychiatrist 2 “Definitely involving the system, involving the family, involving the young person in the transition.” Family Therapist 2
	Education	“I think linking the young person in with other systems, like, for example, education having them linked with college, always helps with the sense of stability in the community and reduces the likelihood that they will reengage in antisocial behaviour. It gives them more support and structure around them, more meaningful activities.” Nurse 2

### Theme 1-Transition Processes and Preparation

Transition processes and preparation was a primary theme identified in the interview data with key professionals involved in young people’s transitions from adolescent secure services to adult services. There were two subthemes: statutory processes and staff experience and infrastructural processes.

#### Sub-Theme: Statutory Processes

Statutory processes were explained as supporting the transition process and staff generally described formal procedure as helpful. All adolescent mental healthcare professionals interviewed described the transition preparation, planning and management consisting of the Care Programme Approach (CPA). CPA is an integral and mandatory process during transition for all young people admitted to adolescent secure services. As part of the CPA young people receive a care plan and care co-ordinator to manage and review this. Transition preparation utilised the CPA and was described as tailored around each young person’s needs and circumstances.

[the CPA is the] formal procedure that supports transitions and that ensures that the way that in which the other units have to interact with us, and then other aspects of transition and transfer are to know on individual basis, depending on need and risk. (Psychologist 1)

The transition process, as explained, was well thought-out and planned with the “*formal procedure”* serving to *“support transitions”.* Staff described early liaison with the identified adult service discussing each case thoroughly and deciding on suitable plans for the young person.

Continuity of care was a feature of this theme with key formulation information pertaining to their individual needs and risks, goals and care preferences described as being available for a young person’s receiving placement. Documentation, storage and accessibility to a young person’s discharge plan was important for ensuring continuity of information transfer to the next placement (e.g. “*a communication folder*”).

each discipline will work with them [the young people] on a discharge plan […] They will have a communication folder that describes who they are, what are the risk concerns, what they like and what they don’t like, what are the things they worked through and what are the things they need to work through, so that’s for them to take along with them. (Psychiatrist 1)

#### Sub-Theme: Staff Experience and Infrastructural Processes

Staff frequently described their experience of the transition process as challenging. Staff indicated that early communication with young people was important (e.g. *“we have that discussion early”* and *“allowing clear communication from the start”*)*.* Some key features this early communication about transition included setting expectations with young people and being clear that they are there to support them to continue any progress made when they return to the community. Young people were informed at admission that they will need to leave adolescent secure services upon turning 18 years old. Therefore, there were an orientation towards transition out of the service and a focus on returning to community placements during early communication between staff and young people.

When young people come to us they are aware that our service is designed for 12–18 year olds. […] So we have that discussion early on so they know that the part of getting support from us is planning how they can maintain their recovery and continue the gains they have made when they are actually part of the community. I think rather than breaking relationships it is allowing clear communication from the start that the work that we can do with the young person is limited. But also reassuring them that there is scope for ongoing support and the earlier that we start that the better. (Psychologist 2)

Staff described challenges relating to commissioning and that *“it’s easier going to an adult hospital”* from a logistical and organisational perspective. Staff also expressed some anxiety about young people returning to community placements for a range of reasons but primarily, the risk of offending and relapse compounded by poor management of these risks in community settings. Staff reported that young people move to forensic adult services despite a supportive community environment being a better fit. The reported commissioning challenges and anxiety amongst mental healthcare professionals related to arranging community placements which may result in young people’s prolonged stay in secure inpatient settings.

It’s very difficult, very challenging. It’s easier going to an adult hospital from a commissioning point of view. […] There’s always a gatekeeping assessment […] Care-coordinators you see them getting nervous when you talk about what placement they have in the community, the forensic, and the offending and the risk point of view but also I think it’s the logistics and sorting it out. (Occupational Therapist)

### Theme 2-Transition Barriers and Challenges

Transition barriers and challenges was a primary theme identified in the staff interview data, with five identified subthemes: therapeutic relationships between key workers and young people, transition timings and relapse of mental health difficulties, transition destination predicting transition outcomes, culture shock on adult wards and readiness to move into adult services.

#### Sub-Theme: Therapeutic Relationships Between Key Workers and Young People

Twenty-five mental healthcare professionals pointed to the difficulty to engage and motivate young people who know from the beginning that they will be moving to other services and subsequently, some find it less meaningful to participate in therapy and/or in educational courses since education is mandatory until their eighteenth birthday. Two of the units included in this research accept patients who are within a 3-month period of their 18th birthday. This cohort of patients transferring to adolescent services just a few months ahead of a forthcoming additional transition to adult services was reported to interfere with establishing a therapeutic relationship and rapport with the patient. Patients are aware of the short duration of stay and subsequently staff felt they were less keen to collaborate with staff.

We have a lot of young people who are admitted to the service relatively late at 17.5 plus and [it] is very difficult to meet their needs when suddenly they are on their 18th birthday and just started to be engaging in formal treatment and make relationships with staff and the pressure is moving them to another service, I think what this inevitably does is extending their stay in hospital. (Psychiatrist 2)

The formal legal processes associated with sectioning a young person under the Mental Health Act were described as *“a stressful experience”* for young people. Clinicians reported conflicting views between the clinician and their patient regarding the outcome of the tribunal which can lead to the relationship becoming *“adversarial”* and straining the rapport built prior to the court proceedings.

I think the tribunal process is important but sometimes it’s very stressful for patients. So, if they appeal against a section, the tribunal is quite a formal thing […]The consultant, the Responsible Clinician, has to argue for the continued detention of the patient and, usually, the patient is asking to take them off the section. So, it can become quite adversarial, it can worsen the relationship between the consultant and the patient. (Psychiatrist 5)

#### Sub-Theme: Transition Timings and Relapse of Mental Health Difficulties

Transitional delays and safeguarding issues in adolescent medium secure units were key features of this subtheme. Thirty-one staff members referred to young people’s difficulty coping with transition delays and the adverse effects of extended periods of waiting. Eight clinicians interviewed mentioned examples of young people being referred to a higher secure unit due to risk escalation meaning they were no longer eligible for lower security services. Transition delays increased uncertainty and anxiety and were described as a frustrating and *“unsettling”* experience for young people. The element of the unknown embedded in these transitions, along with long periods of waiting was reported to trigger violent behaviour and relapse which sometimes led to periods in seclusion.

There’s a lot of people who relapsed they got to the point they were ready for discharge, but it took that long that they ended up back to square one; they relapsed. (Nurse 1)

It was reported that increased support in cases where there was uncertainty around the transition did not always mitigate against relapse. Therefore, transition delays can have deleterious effects on the mental health, social functioning and risk presentation of young people, ultimately extending their period of contact with services.

Well, they [transitions] can be a source of significant anxiety. What I would say is that certainly the uncertainty around what we typically see in the medium secure group is that prior to transitions, peoples’ HoNOSCA scores go up and it’s very unsettling times for young people. And there’s the risk particularly if you’ve got a comorbid mental illness that the stress can make that illness relapse. And we’ve certainly seen that happen occasionally even when people get quite a lot of support around the process. (Psychiatrist 3)

Young people who have turned 18 years old and are waiting for a bed to be released in forensic adult hospitals reportedly experience frustration remaining in child services. They were reported to disengage from education and/or psychology sessions and affect the ward’s dynamics. There was a recognised split between the younger and older adolescents on the ward whilst the former are seen as *“kids”* by the latter.

They [adult secure hospital] can’t tell us when a bed may be available, and we have serious problems for X because he started disengaging from our service. He’s sort of frustrated that he’s still here with the kids, as he reasons. (Psychiatrist 4)

Finding a suitable placement for this complex group of young people was quoted by 32 mental healthcare professionals as a great challenge and waiting for bed availability was cited as a key issue. Interviewees referenced the reluctance of some community placements to accommodate high-risk young people or those who have committed a notorious offence in their communities. Hence, it becomes difficult to return these young people to their local areas where there would be concerns about community safety. Community placements may not have the capacity to manage high-risk and complex forensic histories. It was consistently reported that elapsed time between placements led to significant disruption for the young person resulting in relapse and requiring services to invest resource *“to start again”* to enable the young person to be ready for transition*.*

The hardest thing is probably finding suitable places for the young people and waiting for beds to open up because we had a couple of occasions where we had a bed and they’ve been accepted but the young person had relapsed because it took that long and then ended up not being suitable for the placement anymore, so we had to start again. (Nurse 2)

Young people who have reached 18 years and are still in adolescent services are the oldest on the ward and this often becomes an issue for younger patients who might become subject to bullying and/or assault by their older peers. Therefore, the transition process for the oldest peer to adult services might be expedited due to safeguarding issues.

X who is 18 years, he doesn’t know when he’s going, and we can’t do any preparation. And I imagine that as soon as the bed is available, he will go, because he poses a risk to other young people here. This 18-year-old patient who is bullying the children, he’s also vulnerable too, […] That’s the way we are solving the problem of an 18-year-old bullying children is to get rid of the 18-year-old. (Psychiatrist 8)

#### Sub-Theme: Transition Destination Predicting Transition Outcomes

Transition destination and timing were inextricably linked. Not knowing where young people would be placed following their period in hospital was cited as a key barrier to the transition process and a challenge for therapeutic relationships with young people. The element of uncertainty was reported to increase disengagement and anxiety. Neither party knowing the exact timing and destination of the placement impeded upon smooth endings that might enhance placement stability in the receiving service. Staff reported feeling *“demoralised”* and *“despondent”* at a team-level, mirroring the perceived experience of the young people in their care.

The extended period of time, especially when we don’t have an end date, because the patient becomes very demoralised and destabilised. In fact, the nursing staff, the whole team becomes kind of despondent about, you know, they need to move on, we’ve done as much as we can…and you can have aberrant behaviours re-emerging. (Psychiatrist 5)

Uncertainty in transition destination was compounded by geographical location. Due to the national reach of adolescent medium secure units in England, *“young people from any part of the country”* can be placed there. This was perceived as a barrier to smooth transitions given the difficulty of secure units being connected with services local to young people from across various locations in England. The main difficulties cited were forming initial links and understanding referral criteria. This represents a key need to increase opportunity to connect with other services to determine the most suitable receiving placement for young people, subsequently avoiding further geographic displacement which is a key feature of the service history of this cohort of young people.

I think being a national service is probably the hardest. Because for us to form links locally are really hard and to understand what the criteria for local services are, is very hard. I think that’s the key; the challenge of being a national service and having young people from any part of the country. That’s the biggest problem to getting to smooth transitions really. (Psychiatrist 6)

Risk presentation was reported to affect the transition experience of a young person, namely impacting whether they are discharged to a community placement or hospitalised further, as well as the discharge planning support they receive ahead of time. If risk is manageable, young people are allowed to visit their receiving placement. However, young people presenting with a high level of risk might be transferred to a receiving placement without having visited to meet key workers and familiarise themselves with the new environment prior to transitioning. This was a concern for staff as it risked jeopardising successful transition given the lack of input a young person is given to facilitate the transfer. High risk young people may not *“know the date of transfer”* due to risk of absconding. This serves to increase the uncertainty surrounding transitions out of adolescent secure services and removes the opportunity for them to adequately prepare.

When the risk is high, they will be transferred without these visits, but they will be provided information about the service…They wouldn’t know the date of transfer. (Psychiatrist 1)

This presents a serious concern given that young people posing the highest risk receive less direct support to facilitate a successful transfer, when in fact, they may be most in need of this.

#### Sub-Theme: Culture Shock on Adult Wards

Mental healthcare professionals highlighted that key differences between adult and child and adolescent services present as risk factors to young people’s mental health and risk presentation. More specifically, 24 staff members cited the difficulty young people face coping with the cultural shift from an adolescent inpatient unit to an adult service. Interviewees felt that challenging behaviours such as *“smashing things, shouting, crying”* can be better contained in adolescent services due to a higher staff to patient ratio and greater supervision compared to adult services. Staff reported that young people experienced a *“shock”* upon entering adult services. Despite being described as *“chaotic sometimes”,* adolescent inpatient units were described as a *“nurturing environment”.* Child and adolescent mental health services adopt different care-planning approaches compared with adult mental health services. Hence, transitions for young people become more complicated and young people may not be prepared for the more independent care-model adopted by adult services. This gap between service cultures contributes to poor transitions and mental health outcomes which may subsequently increase risk of relapse or reoffending.

When people go from here to inpatient units, I think it’s a big shock to the patients because they go from this very nurturing environment where even though it is quite chaotic sometimes here, I think it’s much more ordered than an adult unit. So we get a lot of our patients smashing things, shouting, crying; they are very emotional. But we can’t contain that to an environment where is much lower staffing level and there’s much larger groups of patients and the patients are not supervised for much longer. (Psychiatrist 9)

#### Sub-Theme: Readiness to Move into Adult Services

Developmental maturity and emotional readiness were referenced numerous times across the staff interviewed and may represent barriers to successful transition. Those mental healthcare professionals working with young people with learning disabilities and autistic spectrum disorders described this group as being very sensitive to change and less developmentally prepared for transitioning to adult services. Developmental maturity was described as distinct from chronological maturity and emotional readiness. There was a sense that clinical age boundaries for transitioning to adult services were somewhat arbitrary and that chronological maturity was only one element of readiness to transition. An *“intermediate service”* for young adults in transition was thought to have the potential to mitigate against some of the challenges of transitioning to a service with much older adults.

I think it’s a very tricky situation because just on the previous day of their 18^th^ birthday they were just 17 and the day after they become adults, is there any change overnight? I don’t think so. It’s a gradual process. I would think there should be an intermediate service, like service for 18–25 s. (Psychologist 3)

The shift in social status and hierarchy in the receiving placement in adult services can become very complex for the young person. There is a concurrent pressure to adjust to a new environment and also accept a new assigned status as younger and inferior, affecting their placement stability and perceived safety. Transition to adult placements were perceived by staff to be a *“terrifying”* experience for young people given the loss of key relationships with staff and peers, as well as navigating a new social and personal identity in relation to a new adult-oriented environment.

They get so frightened and cowered… then you’re going into an adult service where there’s going to be people in their 30 and 40s and maybe in their 50 s on the same ward. It must be terrifying for the young people and terrifying for the families. (Social Worker 1)

### Theme 3-Transition Success Factors

Transition success factors was a primary theme identified in the staff interview data, with three identified subthemes: community integration, family and young person involvement and structure as a protective factor to transitions.

#### Sub-Theme: Community Integration

Involving local services from the young person’s catchment area was identified as a protective factor, aiding successful transitions. Twenty-four mental healthcare professionals mentioned that early communication with a key contact from local services, as well as a consistent identified care-coordinator facilitated transitions.

If you got a good care-coordinator and if you are linked with local services from the onset, that really helps. (Psychologist 4)

Twenty-three mental healthcare professionals identified the Mental Health Act as a protective factor for young people’s continuity of care. It was described as both *“useful”* and playing a *“positive role”* in the transition process*.*

The Mental Health Act is useful—you got to discharge them [young people] and you still have treatment obligations carried out, which means they are assured to get follow up. (Psychiatrist 7)

The Aftercare section ensures that young people will be provided mental health service provision. Staff spoke about Extended 117 leave from a rights-based perspective and felt it supported transitions to community placements by preserving young people’s entitlement to follow-up.

All the young people are under the Mental Health Act so […] depending on which section they are on […]they all have to be discharged from a section of the Mental Health Act conditionally. Most of them have got an entitlement to Aftercare under Section 117. I guess on the whole the Mental Health Act plays a positive role, probably. It gives young people that are very restricted and being looked after, in some sense, it gives them some protections i[…] some rights and it does give them some follow-up entitlement. (Nurse 3)

#### Sub-Theme: Family and Young Person Involvement

Family involvement was described as a protective factor to successful transitions amongst mental healthcare professionals interviewed. A systemic approach to transitions was described as essential by 25 staff members, enabling the young person and their family to be involved and informed through the process. When asked about facilitators to successful transitions out of adolescent secure services, one interviewee shared:

Definitely involving the system, involving the family, involving the young person in the transition. (Family Therapist 2)

Staff felt that involving families/carers of young people particularly aided their reintegration into the community. Amongst staff interviewed, 12 mentioned formal processes in place to engage families of young people in their care. These ranged from clinical interventions, to organisational initiatives such as open days and welcome meetings with the aim of providing *“more understanding and more help”.* Interviewees largely felt that ensuring families are well-informed and involved in the transition process facilitated successful transition.

We do have family therapy, we do welcome meetings, we do open days for the parents to get them engaged and to give them more understanding and more help. (Psychiatrist 2)

#### Sub-Theme: Structure as a Protective Factor to Transitions

Successful transitions to the community were described by staff as having *“a sense of stability”.* Linking young people with education services, such as college, was thought to promote this sense of stability and thus contribute to a reduced likelihood of reengagement in antisocial behaviour. Education in the community was believed to provide both *“support”* and *“structure”* through allowing young people to engage with *“meaningful activities”,* which was deemed to be a core characteristic of successful transition.

I think linking the young person in with other systems, like, for example, education having them linked with college, always helps with the sense of stability in the community and reduces the likelihood that they will reengage in antisocial behaviour. It gives them more support and structure around them, more meaningful activities. (Nurse 2)

Thirteen interviewees expressed concern about discontinued structure and non-compulsory education involved in the transition from child to adult services. *“Keeping busy”* was thought to be a protective factor reducing challenging behaviours and maintaining a positive unit environment in adolescent inpatient facilities. Staff reported that young people *“struggle”* with the lack of structure upon transition to adult services.

In forensic child and adolescent services, we have much more structured days because we have college they have to go to and we have lot more activities on. We try to keep them busy because adult patients can sit around all day doing nothing and go to bed again and carry on doing that and if the children are let to do that, you will have chaos on your arms. They will just fight, argue, it can be very difficult, so we try to keep them busy. I think when they move to adult inpatient units, they really struggle. (Psychiatrist 5)

## Discussion

The qualitative interviews with mental healthcare professionals elicited key issues about the transitions of young people from adolescent medium secure units to adult services. Key findings from the study pertained to service culture shifts and ending therapeutic relationships, risk presentation and community accommodation, bed delays and mental health deterioration, geographical location of services and transitional readiness to move onto adult services. The implications deriving from the findings can inform policy and practice and add important knowledge to the transition literature about healthcare providers’ experiences.

### Difference in Services’ Cultures

Mental healthcare professionals highlighted the key differences between forensic child and adolescent inpatient services and adult services as risk factors to young people’s mental health and risk presentation. Adult services focus on an isolated model for the young person and moving to an adult service requires adapting to a different culture in addition to adjusting to the new service’s infrastructural procedures (Leavey 2009). Young people with autism and learning disabilities appeared the most disadvantaged, as they were experiencing isolation on adult wards. Additionally, there is a shift in parental involvement and responsibility in adult services. The responsible clinicians from adult services interviewed did not report on building trusting relationships with parents or involving them directly in the young people’s care.

Multi-agency/service collaboration becomes challenging, heightened by the gap between child and adolescent mental services and adult mental health services (Davis 2003). Child and adolescent mental health services adopt different care-planning approaches than those used by adult mental health services (Singh et al. 2005). Hence, transitions for young people become more complicated and young people may not be prepared for the more independent care-model adopted by adult services. This gap between services contributes to poor transitions and mental health outcomes and subsequently may increase the risk for reoffending (Livanou et al. 2017).

Young people transitioning back to their communities have the poorest outcomes if they do not receive adequate care (Wright et al. 2016). Research has evidenced that approximately 68% of children who return to community reoffend within one year (Goodfellow et al. 2015). Chitsabesan et al. (2006) found that young offenders released from custody encountered major challenges and their mental health symptoms worsened in the community. Service provision declines once young people enter adulthood and they often lose the support they were eligible for as children (Davis 2003). Therefore, community discharge needs to be a continually reviewed process effectively using extension 117 leave of the Mental Health Act. Both child and adolescent and adult mental healthcare professionals can facilitate the process by providing ongoing support to the young people and their families.

### Ending Therapeutic Relationships

Mental healthcare professionals discussed the challenges of ending relationships with staff for young people and the difficulties that can emerge during periods of transition. Young people might encounter a greater difficulty in letting go considering their childhoods are underlined by loss and/or trauma (Broad et al. 2017). As Lindgren et al. (2014) argued, young people feel ambivalent towards their prospective transitions due to the uncertainty of moving to a new service where they have to start all over again in building relationships with staff. Young people discharged from forensic services are less likely to establish trusting relationships with authorities, as they have received inconsistent care from parents and services in the past (Board 2016).

### Complexity of Needs and Risk Presentation

Sixty-seven percent of mental healthcare professionals referred to those young people transitioning from adolescent secure services to community placements as being a highly unwanted group considering the stigma and severity of the index offence. These young people present with very challenging behaviours that cannot be managed in community placements due to lack of appropriate training and possibly anxiety to manage high-risk young people with comorbid mental health problems (Belling et al. 2011). Furthermore, the stigma of the offence remains high and, therefore, community services may not accept young people presenting with risky behaviour upon discharge from medium secure units based on the crime severity committed in the past (Chitsabesan et al. 2006).

However, risk is a concerning factor along with past forensic history. There are cases where the young person cannot return to their communities as there is a need to protect the victims as well as the young person. This exacerbates the experience of disenfranchisement for young people who offend. In other cases, young people might move to a community placement, spending only a short amount of time there before returning back to adolescent medium secure units because they could not manage independent living. Accordingly, both community services and the service users feel incapable of moving on. Adult services are less likely to adopt trauma-informed care and gain a developmental understanding on young people’s adverse experiences, therefore they tend to exclude and/or reject them (Wright et al. 2016).

These dynamics result in more confusion for the young people and their clinicians who are called to identify a new placement for the young person. It could be an extremely frustrating experience for the young people, as by first moving to the community, they were told by their child responsible clinician that they were ready to move on. However, they are ‘returned’ to the forensic services due to their incompetency of being independent in ‘one night.’ This transition has a regressive character that may have a tremendous impact on their mental health and their future risk presentation and could be seen as a ‘double punishment’ to the services’ high expectations (Wright et al. 2016).

### Young People in Adult Placements

The existing literature along with NICE guidelines—evidence-based and quality standards recommendations about clinical practice by independent groups of professionals and lay members of the public in the UK—underscores that young people should not be placed in adult mental health hospitals when they are below 18 years. The risk of placing adolescents in adult placements pertains to young people being vulnerable and prone to bullying by much older peers, and their needs can be overlooked. Even young people who are legally adults encounter a number of difficulties when placed on adult wards and/or placements. It must be emphasised that these young people are leaving adolescent secure services units, where they are the oldest amongst their peers, and are transferring to new institutions, where they are the youngest amongst much older peers in their late 50 and 60 s. This shift in social status and hierarchy in the receiving institution in which they are placed can become very complex for the young person. Concurrently, they have to adjust to a new environment and also accept a new assigned status that is inferior to the one they had previously in the institution in which they felt safer. Transition to adult placements can become a terrifying experience where they lose relationships with key figures, peers, and their social and personal identity is shaken.

### Transitional Delays

Waiting time to be discharged to adult forensic services was the most quoted theme in the interviews with mental healthcare professionals. Transitional delays accounted for deterioration of mental health problems, trigger violent behaviour and relapses on the ward, disengagement from therapeutic relationships and education, isolation, bullying and safeguarding issues (McNamara et al. 2017). Transitional delays mostly accounted for organisational and infrastructural weakness of current services (McNamara et al. 2017). Both child and adolescent and adult mental healthcare professionals had to overcome presenting challenges and facilitate transitions to services. Young people involved in aggressive behaviour towards staff members and/or peers were separated and put into seclusion where their symptoms seemed to aggregate. The level of risk therefore changed during delayed transitions and mental healthcare professionals had to identify new placements because community settings would not take young people with recent violent incidents who were perceived unready to deal with the challenges in the community.

### Transition Readiness

The age boundary policy on young people moving to adult services once they turn 18 years has been discussed multiple times in the extant literature (Singh et al. 2010). Age criteria should be flexible and pragmatic in terms of meeting young people’s needs and consider developmental maturity, chronological maturity and emotional readiness. At least four of the services reported some flexibility with keeping service users until their 19th birthday. One service pointed out that they were not allowed to keep young people until they turn 19 and they had to expedite the transition process to meet the commissioning requirements. Funding pressure can be particularly problematic during transition times and may often impact transition outcomes negatively by moving young people to less suitable placements which do not meet their needs.

Young people moving to forensic adult inpatient services are not assessed by the identified receiving service until the young person has turned 18 years. This becomes more of an issue when it is decided that the young person is to step down security levels-to move from the medium secure unit to an adult low secure. If the assessment process is delayed, there is uncertainty surrounding the young person’s prospective transition and the young person cannot be informed about the date of moving and/or visit the placement. Accordingly, this creates another barrier to the therapeutic relationship between the child responsible clinician and the young person. The responsible clinician can be perceived as accountable for the young person’s situation and their therapeutic relationship may be damaged.

### Limitations

This study only included experiences of mental healthcare professionals from national medium secure units and selected adult services based on the transition destination of the young people. More research aiming for a wider sample of healthcare professionals, young people and a greater pool across services such as low secure units and PICUs, needs to garner service experiences. It is possible that other service providers hold different views and attitudes towards transitions and implement different approaches to prepare young people for adult services and community discharge. Additionally, as certain healthcare professions were underrepresented, including occupational therapists and nurses due to their high clinical caseloads and time restraints, it is likely that these underrepresented groups may employ different approaches to transition preparation. However, the current sample was diverse in terms of other professional types and geographical location. Follow-up studies with young people at different times post-discharge would help to understand and reflect on their experiences.

## Conclusions

This was the first national study focussing on the experiences of mental healthcare professionals involved in transitions from adolescent forensic hospitals to adult mental health services and community settings. Mental healthcare professionals across a wide range of forensic mental health settings shared their views on transitions and reported the organisational and infrastructural weaknesses and challenges in the current system. Interviewing key workers from child and adult services was very useful in generating and corroborating key themes that should be central to policy decisions and clinical practice. Conflicting views between child and adolescent and adult mental healthcare professionals need to be considered in refining current multiagency approaches. Furthermore, establishing transition teams in adolescent secure services will help to implement a ‘young person friendly approach’ to ensure standardised and uniform processes. Transition guidelines built on evidence-based research need to be developed across adolescent secure services to improve current practice during transition periods. These practice/clinical guidelines should be tailored around each service’s and young person’s individualised needs considering risk presentation and protective factors.

A standardised national database for young people in adolescent secure services that is regularly updated would help to build an understanding of this group’s needs up to date. Transition outcomes and preparation need to be captured within this database where future research should study the link between service involvement and transition outcomes.

## References

[CR1] Begoray D, Banister E, Mills A, Durepos G (2010). Reflexivity. Encyclopedia of case study research.

[CR2] Belling R, Whittock M, McLaren S, Burns T, Catty J, Jones I, Rose D, Wykes T, ECHO Group (2011). Achieving continuity of care: Facilitators and barriers in community mental health teams. Implementation Science.

[CR3] Board YJ (2016). Participation strategy: Giving young people a voice in youth justice.

[CR4] Braun V, Clarke V (2006). Using thematic analysis in psychology. Qualitative Research in Psychology.

[CR5] Broad KL, Sandhu VK, Sunderji N, Charach A (2017). Youth experiences of transition from child mental health services to adult mental health services: A qualitative thematic synthesis. BMC Psychiatry.

[CR6] Campbell S, Abbott S, Simpson A (2014). Young offenders with mental health problems in transition. The Journal of Mental Health Training, Education and Practice..

[CR150] Care Quality Commission (2019). Segregation in mental health wards for children and young people and in wards for people with a learning disability or autism. 2019. Retrieved 1 October, 2019, from www.cqc.org.uk/sites/default/files/20190626_rssinterimreport_full.pdf.

[CR7] Chitsabesan P, Kroll L, Bailey SUE, Kenning C, Sneider S, MacDonald W, Theodosiou L (2006). Mental health needs of young offenders in custody and in the community. The British Journal of Psychiatry.

[CR8] Davis M (2003). Addressing the needs of youth in transition to adulthood. Administration and Policy in Mental Health and Mental Health Services Research.

[CR9] Given LM (2008). The Sage encyclopedia of qualitative research methods.

[CR10] Goodfellow, P., Wilkinson, S., Hazel, N., Bateman, T., Liddle, M., Wright, S., & Factor, F. (2015). Effective resettlement of young people: Lessons from Beyond Youth Custody. Retrieved frrom http://irep.ntu.ac.uk/id/eprint/32949/1/PubSub10329_Goodfellow.pdf. Accessed 10 May 2020

[CR11] Hales H, Holt C, Delmage E, Lengua C (2019). What next for adolescent forensic mental health research?. Criminal Behaviour and Mental Health.

[CR12] Hollins L (2019). Review of restraint, prolonged seclusion and segregation for people with a mental health problem, a learning disability or autism. International Journal of Positive Behavioural Support.

[CR13] Leavey JE (2009). Youth experiences of living with mental health problems: Emergence, loss, adaptation and recovery (ELAR). Canadian Journal of Community Mental Health.

[CR14] Lindgren E, Söderberg S, Skär L (2014). Managing transition with support: Experiences of transition from child and adolescent psychiatry to general adult psychiatry narrated by young adults and relatives. Psychiatry Journal.

[CR15] Livanou MI, Furtado V, Singh SP (2017). Mentally disordered young offenders in transition from child and adolescent to adult mental health services across England and Wales. Journal of Forensic Practice.

[CR16] Livanou M, Singh SP, Liapi F, Furtado V (2020). Mapping transitional care pathways among young people discharged from adolescent forensic medium secure units in England. Medicine, Science and the Law.

[CR17] McNamara N, Coyne I, Ford T, Paul M, Singh S, McNicholas F (2017). Exploring social identity change during mental healthcare transition. European Journal of Social Psychology.

[CR18] Paul M, Ford T, Kramer T, Islam Z, Harley K, Singh SP (2013). Transfers and transitions between child and adult mental health services. The British Journal of Psychiatry.

[CR19] Signorini G, Singh SP, Marsanic VB, Dieleman G, Dodig-Ćurković K, Franic T, Gerritsen SE, Griffin J, Maras A, McNicholas F, O’Hara L (2018). The interface between child/adolescent and adult mental health services: Results from a European 28-country survey. European Child and Adolescent Psychiatry.

[CR20] Singh SP, Evans N, Sireling L, Stuart H (2005). Mind the gap: The interface between child and adult mental health services. Psychiatric Bulletin.

[CR21] Singh SP, Paul M, Ford T, Kramer T, Weaver T (2008). Transitions of care from child and adolescent mental health services to adult mental health services (TRACK study): A study of protocols in Greater London. BMC Health Services Research.

[CR22] Singh SP, Paul M, Ford T, Kramer T, Weaver T, McLaren SS, Hovish K, Islam Z, Belling R, White S (2010). Process, outcome and experience of transition from child to adult mental healthcare: Multiperspective study. The British Journal of Psychiatry.

[CR23] Wright, S., Liddle, M., & Goodfellow, P. (2016). Young offenders and trauma: Experience and impact: A practitioner’s guide. Retrieved July 10, 2020, from http://irep.ntu.ac.uk/id/eprint/32944/1/PubSub10327_Goodfellow.pdf

